# scMoresDB: A comprehensive database of single-cell multi-omics data for human respiratory system

**DOI:** 10.1016/j.isci.2024.109567

**Published:** 2024-03-26

**Authors:** Kang Chen, Yutong Han, Yanni Wang, Dingli Zhou, Fanjie Wu, Wenhao Cai, Shikang Zheng, Qinyuan Xiao, Haiyue Zhang, Weizhong Li

**Affiliations:** 1 Zhongshan School of Medicine, Sun Yat-sen University, Guangzhou 510080, Guangdong Province, China; 2 Key Laboratory of Tropical Disease Control of Ministry of Education, Sun Yat-Sen University, Guangzhou 510080, Guangdong Province, China; 3 Center for Precision Medicine, Sun Yat-sen University, Guangzhou 510080, Guangdong Province, China

**Keywords:** Body system, Human, Biological database, Data processing in systems biology, Transcriptomics

## Abstract

The human respiratory system is a complex and important system that can suffer a variety of diseases. Single-cell sequencing technologies, applied in many respiratory disease studies, have enhanced our ability in characterizing molecular and phenotypic features at a single-cell resolution. The exponentially increasing data from these studies have consequently led to difficulties in data sharing and analysis. Here, we present scMoresDB, a single-cell multi-omics database platform with extensive omics types tailored for human respiratory diseases. scMoresDB re-analyzes single-cell multi-omics datasets, providing a user-friendly interface with cross-omics search capabilities, interactive visualizations, and analytical tools for comprehensive data sharing and integrative analysis. Our example applications highlight the potential significance of BSG receptor in SARS-CoV-2 infection as well as the involvement of HHIP and TGFB2 in the development and progression of chronic obstructive pulmonary disease. scMoresDB significantly increases accessibility and utility of single-cell data relevant to human respiratory system and associated diseases.

## Highlights

• A single-cell database with extensive omics types for human respiratory diseases
• It gains advance in data coverage, database functionality, and research applications
• Its visualizations suggest the significance of BSG receptor in SARS-CoV-2 infection
• A case study shows the HHIP/TGFB2 involvement in COPD development and progression

## Graphical abstract

**Figure:**
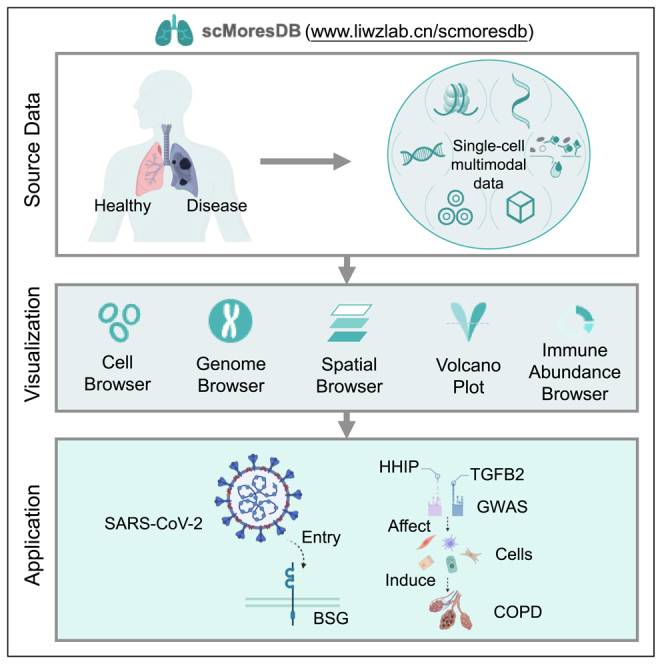

## Introduction

The human respiratory system is a complex and important system that can suffer a variety of diseases. Notably, as reported by the World Health Organization (www.who.org), coronavirus disease 2019 (COVID-19) has caused nearly 7 million fatalities worldwide, profoundly impacting both public health and global economic stability. Concurrently, in 2019, other respiratory diseases such as chronic obstructive pulmonary disease (COPD), lower respiratory tract infections, and lung cancers accounted for approximately 7.7 million deaths, representing 13.9% of total deaths of the year.^1^ Despite substantial clinical research has focused on the treatment and prevention of human respiratory diseases, the mechanisms of many respiratory diseases remain inadequately elucidated.

In recent times, the mechanisms of occurrence and development of respiratory diseases have been better deciphered through next-generation sequencing (NGS). During the early phase of COVID-19 pandemic, NGS facilitated the acquisition of full-length genomic sequence of severe acute respiratory syndrome coronavirus 2 (SARS-CoV-2), offering initial insights into the virus.^2^^,^^3^ Subsequently, the genomic information played a pivotal role in the diagnosis of disease and the development of vaccines, drugs, and therapies. Particularly, the emergence of single-cell sequencing technologies, including single-cell RNA sequencing (scRNA-seq),^4^ single-cell assay for transposase-accessible chromatin followed by sequencing (scATAC-seq),^5^^,^^6^ single-cell T cell receptor sequencing (scTCR-seq),^7^ and cellular indexing of transcriptome and epitope by sequencing (CITE-seq),^8^ has significantly enhanced our capability to precisely characterize molecular and phenotypic features in respiratory diseases at a single-cell resolution. This advancement has facilitated the identification of the novel biomarkers, the types, states, and fates of cells, as well as the interactions between cells, consequently paving the way for innovative therapeutic interventions and treatments for respiratory illnesses.^9^^,^^10^^,^^11^^,^^12^

Single-cell studies have led to a surge of single-cell multi-omics data, which are usually stored in various public databases. While several multi-omics databases such as SC2diseases,^13^ Aging Atlas,^14^ HTCA,^15^ and SCLC-CellMiner^16^ and respiratory disease databases like LCMD,^17^ REALGAR,^18^ LDGDB,^19^ LungMAP,^20^ and LGEA^21^^,^^22^ have been established, few incorporates single-cell multi-omics sequencing data. Additionally, these databases contain limited amount of data for respiratory diseases. The utilization and analysis of these resources requires a significant investment of time and expertise. Comprehensive integration and effective utilization of the molecular data resources for human respiratory system are urgently needed for both basic researches and clinical applications. Hence, providing a user-friendly, centralized, and comprehensive data portal to facilitate access to single-cell multi-omics data of human respiratory system is crucial.

**Figure 1:**
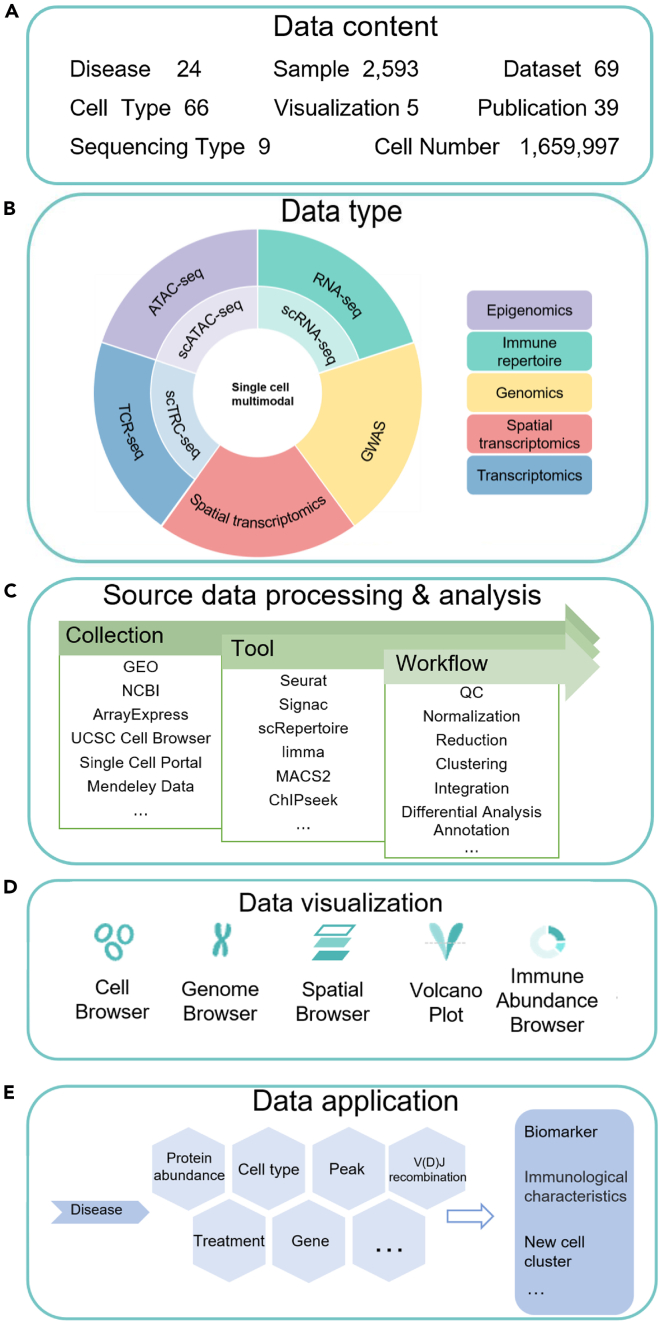
The design layers of scMoresDB (A) Data content and statistics of scMoresDB. (B) Omics data types in scMoresDB. (C) Data sources, tools, and brief workflows for data processing. (D) Data visualization tools. (E) The database applications of scMoresDB.

In this study, we developed scMoresDB (www.liwzlab.cn/scmoresdb/), a novel database serving as a centralized hub for comprehensive single-cell multi-omics data for human respiratory system and associated diseases. scMoresDB encompasses 9 types of molecular omics data from 69 datasets, covering approximately 1.66 million cells derived from 66 cell types and 2,593 respiratory samples in diverse conditions including healthy, aging, smoking, and 24 respiratory diseases (Figures 1A and 1B). Notably, single-cell multi-omics datasets from different sources were collected and reanalyzed using various tools (Figure 1C) and can be interactively visualized by five distinct viewers (Figure 1D). We further conducted case studies in COPD and COVID-19 using scMoresDB to unveil novel insights and research clues, illustrating its potential value (Figure 1E).

## Results

**Figure 2:**
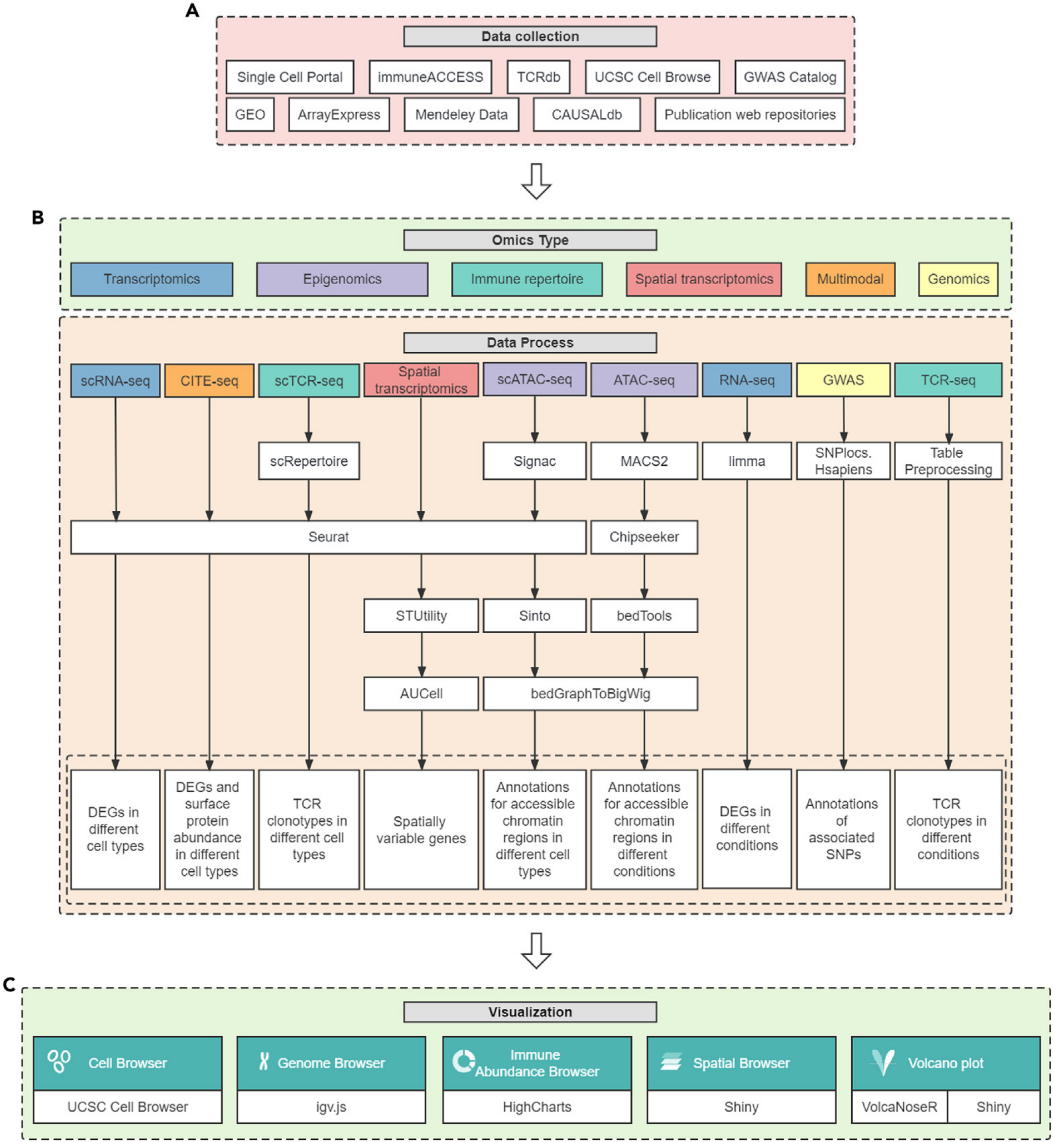
The flow chart of data collection, processing, analysis, and visualization in details (A) The collection of source data. (B) The data processing and analysis for multi-omics types. (C) The construction of 5 visualization tools. See also Table S1.

The homepage of scMoresDB displays the overviews of data statistics, disease data module, multi-omics data module, and visualization tool module. In the disease data module, the disease classification and the cell type annotation adopted the controlled vocabularies of Disease Ontology^23^ and Cell Ontology,^24^ respectively. In the multi-omics module, the data types include single-cell and bulk RNA sequencing (RNA-seq) for transcriptomics, assay for transposase-accessible chromatin followed by sequencing (ATAC-seq) for epigenomics, T cell receptor sequencing (TCR-seq) for immune repertoire, spatial transcriptomics sequencing for spatial omics, and CITE-seq for multi-modal omics. The visualization tool module offers Cell Browser, Genome Browser, Immune Abundance Browser, Spatial Browser, and volcano plot.^25^ The data resources for scMoresDB are listed with statistical information in Table 1, and the flow chart of data collection, processing, analysis, and visualization is illustrated in Figure 2. More details about the source data integration and the web implementation can be found in STAR Methods.

**Table 1:** Statistics of data resource for scMoresDB

Modality	Data statistic	Key information in search module	Visualization type
Single-cell transcriptomics	6 disease types, 154 samples, 40 cell types, 872,591 cells	DEGs in different cell types	Cell Browser
Bulk transcriptomics	8 disease types, 1,477 samples, 60,443 DEGs	DEGs in different conditions	Volcano plot
Single-cell epigenomics	1 disease type, 6 samples, 45 cell types, 19,360 cells	Annotations for accessible chromatin regions in different cell types	Cell Browser, Genome Browser
Bulk epigenomics	1 disease type, 1 sample, 4 tissue types	Annotations for accessible chromatin regions in different conditions	Genome Browser
Single-cell immune repertoire sequencing	2 disease types, 84 samples, 86,361 cells	TCR clonotypes in different cell types	Cell Browser, circos plot
Bulk immune repertoire sequencing	9 disease types, 1,233 samples, 6 tissue types	TCR clonotypes in different conditions	Circos plot
Single-cell multimodal	1 disease type, 136 samples, 20 cell types, 681,730 cells	DEGs and surface protein abundance in different cell types	Cell Browser
Spatial transcriptomics	1 disease type, 8 samples, 11,969 spots	Spatially variable genes	Spatial Browser
GWAS	87 traits, 323,928 associated SNPs	Annotation of associated SNPs	Genome Browser

### Multi-omics data search and browse

**Figure 3:**
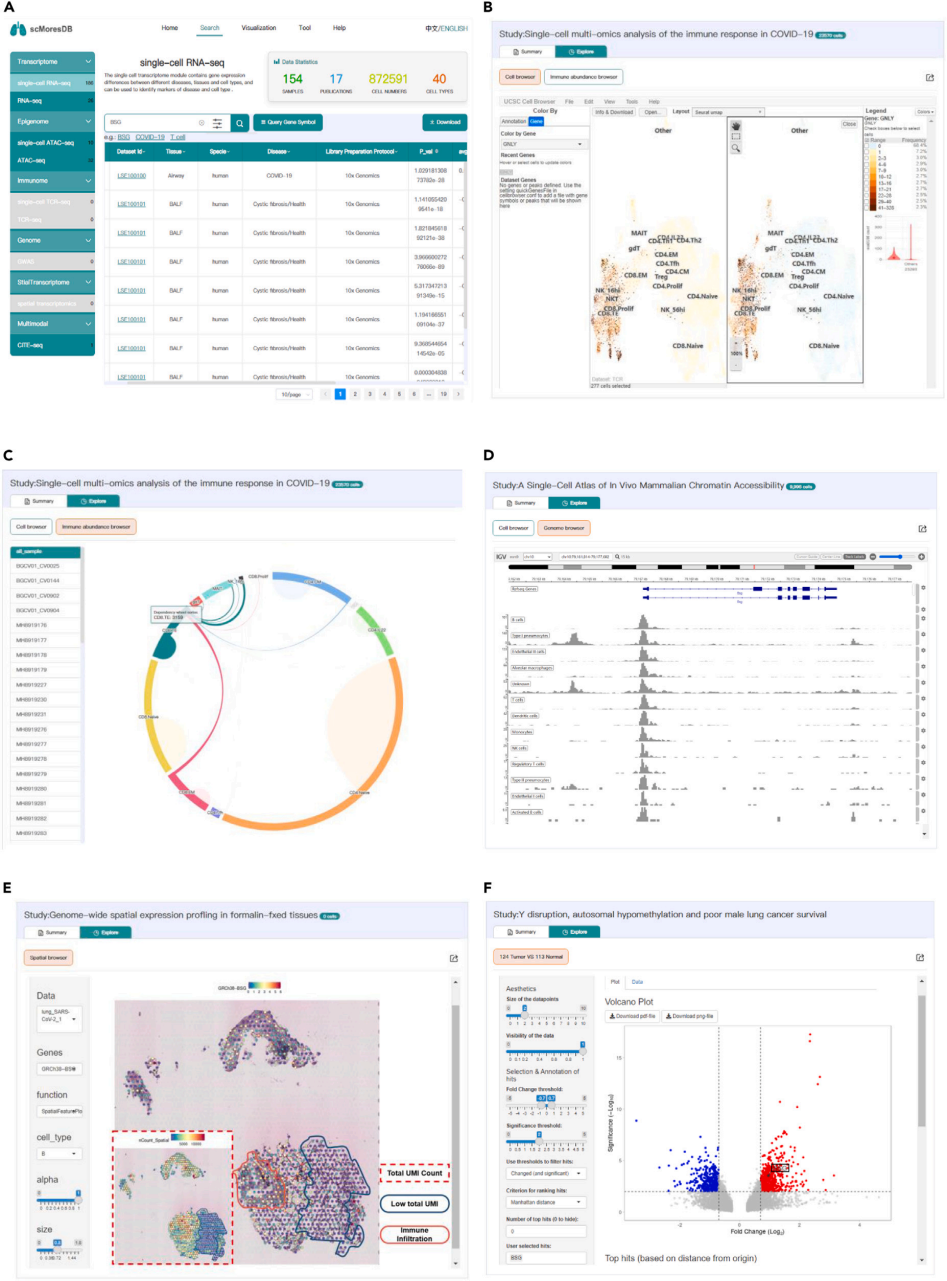
The visualizations of data search and browsers in scMoresDB (A) The result table page of a multi-omics searching for the BSG gene. (B) The Cell Browser displays the UMAPs for a multi-omics dataset of COVID-19. The main layout view is divided into two vertical panels. The cells on the left panel are colored based on the frequency metadata, which represents the number of cells with the shared TCR clonotype; the cells on the right panel are colored based on the gene expression of GNLY. (C) The Immune Abundance Browser displays the sharing of TCR clonotypes among different cell types for the same COVID-19 dataset. The left navigation menu selects “all_sample” to display TCR clonotypes that are shared by cell types in all the samples. The right-side image highlights the effector CD8 cells that share TCRs with other cell types. (D) The Genome Browser visualizes the lung scATAC-seq dataset from a mouse. The interface shows open chromatin regions around the BSG gene in various cell types, including macrophages, T cells, dendritic cells, monocytes, NK cells, regulatory T cells, type II pneumocytes, endothelial cells, and activated B cells. (E) The Spatial Browser displays a spatial transcriptomic dataset from COVID-19 bronchioles and alveolar tissues. The spots in the main view are colored based on the expression of the BSG gene, and the spots in the bottom-left dash box are shaded based on the total number of UMIs in each spot. (F) The volcano plot displays the DEGs for an RNA-seq dataset from lung cancer tumor and non-tumor tissues. The red plots indicate significantly upregulated genes in tumor tissues, while the blue plots show significantly upregulated genes in non-tumor tissues. The black box in the volcano figure illustrates the significantly upregulated expression of the BSG gene.

The search page enables to navigate data in either gene-, cell type-, or disease-centric manner through data searching and filtering (Figure 3A). The datasets from different publications were re-analyzed, generating corresponding tables that contain key information about respective omics. The data selection menu on the left side of search page lists the multi-omics data types for user’s selection as well as the number of match items for each omics type (Figure 3A left menu). Data statistics for the numbers of samples, publications, cells, and cell types is illustrated on the right-top corner of search page. The search box enables user to submit keywords, such as gene symbols and metadata attributes, to search data within a single omics or across multi-omics, and subsequently to filter cell types, tissue types, and disease types (Figure 3A search box). Resulting tables from searching or browsing offer key information of differentially expressed genes (DEGs) from diverse conditions and different cell types for transcriptomics, T cell receptor (TCR) clonotypes and their frequency for immune repertoire, and local usage annotation for epigenomics. Particularly, the resulting table for spatial omics and multi-modal omics presents essential information about spatially variable genes and DEGs plus surface proteins. The dataset IDs link to the data summary and visualization for the dataset. The downloading link on the top-right corner of the result page eases raw data retrieval from external resources.

Users can also browse disease categories from homepage and search for healthy or disease-associated multi-omics data. The diseases are categorized based on Disease Ontology and presented in a hierarchical tree structure for easy navigation. 24 major respiratory diseases are included, such as small-cell lung carcinoma, lung adenocarcinoma, and lung squamous cell carcinoma for lung cancers; COVID-19, Klebsiella pneumonia, influenza, cytomegalovirus infection, and tuberculosis for respiratory tract infectious diseases; and COPD, asthma, idiopathic pulmonary fibrosis, hypersensitivity pneumonitis, cystic fibrosis, extrinsic allergic alveolitis, and interstitial lung disease (ILD) for lower respiratory tract diseases.

### Analysis applications by data visualization tools

The datasets of different omics can be visualized by the UCSC Cell Browser for scRNA-seq, scATAC-seq, scTCR-seq, and CITE-seq, the IGV Genome Browser^26^ for ATAC-seq, scATAC-seq, and genome-wide association study (GWAS), and the Spatial Browser for spatial transcriptomics. The differential gene expression of RNA-seq datasets and the immune abundance of TCR-seq datasets have been re-analyzed and can be plotted in the interactive volcano view as well as the Immune Abundance Browser.

In the UCSC Cell Browser (Figure 3B), cells can be colored according to the provided annotation (e.g., cell types and TCR abundance). Additionally, cells can also be colored by gene expression, scATAC-seq peaks, and CITE-seq surface protein abundance. For instance, the frequency of shared TCR clonotypes per cell from the scTCR-seq dataset (Figure 3B left) can be queried along with the expression level of the granulysin (GNLY) gene from the corresponding scRNA dataset (Figure 3B right). A high proportion of clonal T cells are shown among T cells that express high levels of GNLY, particularly among effector CD8^+^ T cells.

The Immune Abundance Browser (Figure 3C) allows visualization for the pairing of different clonotypic VJ genes in TCR-seq datasets and for the sharing of clonality between different cell types and samples in scTCR-seq datasets. For example, the Immune Abundance Browser of the scTCR-seq data displays the shared TCR clonotypes which are identified from effector T cell subtypes (Figure 3C). This observation is consistent with Ji-Yuan Zhang’s research,^27^ suggesting a potential state transformation in effector T cells.

The Genome Browser (Figure 3D) visualizes enrichment of peaks for different cell types in scATAC-seq datasets and for different samples in ATAC-seq datasets. For instance, the Genome Browser for the mouse lung scATAC-seq dataset demonstrates that the open regions of chromatin are enriched in the promoters of basigin (BSG) gene in various immune cells, epithelial cells, and endothelial cells (Figure 3D). BSG is one of the receptors of SARS-CoV-2 spike protein.^28^ This finding suggests the contribution of BSG in SARS-CoV-2 infection via infecting lung epithelial cells and spreading to immune and endothelial cells, consistent with the discovery of an RNA-seq study by Radzikowska et al.^29^

The Spatial Browser enables to color spots on tissue sections using information on gene expression and clusters. The spatial spots for a small spatial transcriptomics sequencing dataset of bronchial and alveolar tissue derived from COVID-19 patients (Figure 3E) display highly expressed BSG in many regions of both bronchial and alveolar areas, suggesting the pivotal role of BSG in SARS-CoV-2 infection process. Particularly, the BSG expression in the alveolar epithelial region (Figure 3E solid blue circle) is lower than that in the regions with immune infiltration (Figure 3E solid red circle). However, this may be attributed to relatively fewer counts of unique molecular identifiers (UMIs) detected in the alveolar region. The spots detected by the 10X Visium assay do not reach single-cell precision, and the composition of single-layered epithelial cells and multiple cavities in the alveolar region may lead to the relatively fewer counts of UMIs detected by each spot in this area (Figure 3E dash red box). Hence, we can see a correlation trend between the total number of UMIs per spot and the expression level of BSG, which also supports our interpretation described previously.

Finally, the volcano plot (Figure 3F) allows us to visualize DEGs. Thresholds can be adjusted to identify genes that differ significantly compared to control experiments. Through the volcano plot (Figure 3F), a prominent upregulation of BSG is detected in tumor cells. Interestingly, several clinical studies reported that patients with lung cancer were more susceptible to SARS-CoV-2 infection and more likely to develop severe symptoms of COVID-19. This suggests a possible correlation between the BSG expression and the susceptibility of lung cancer patients to SARS-CoV-2 infection.

### A case study in COPD using scMoresDB

The application of scMoresDB enables users to access crucial information concerning respiratory diseases, encompassing disease-associated surface protein abundance, cell types, chromosomal accessibility regions peak, V(D)J rearrangements of TCR, experimental treatments, and gene expressions (Figure 1E). The output information can infer disease-associated biomarkers, immune characteristics, novel cell types, and more. In this study, the utility of scMoresDB in multi-omics research for lung diseases is demonstrated using COPD as an example to explore the heterogeneity of pathogenic mutations at a single-cell resolution. COPD, a pervasive and slowly progressing respiratory ailment, leads to persistent airflow restriction due to irreversible chronic inflammation, ranking as the third leading cause of global mortality.^30^ While GWASs have pinpointed genomic variants associated with heightened COPD risk, the precise mechanisms by which these loci influence disease pathogenesis remain elusive.

**Figure 4:**
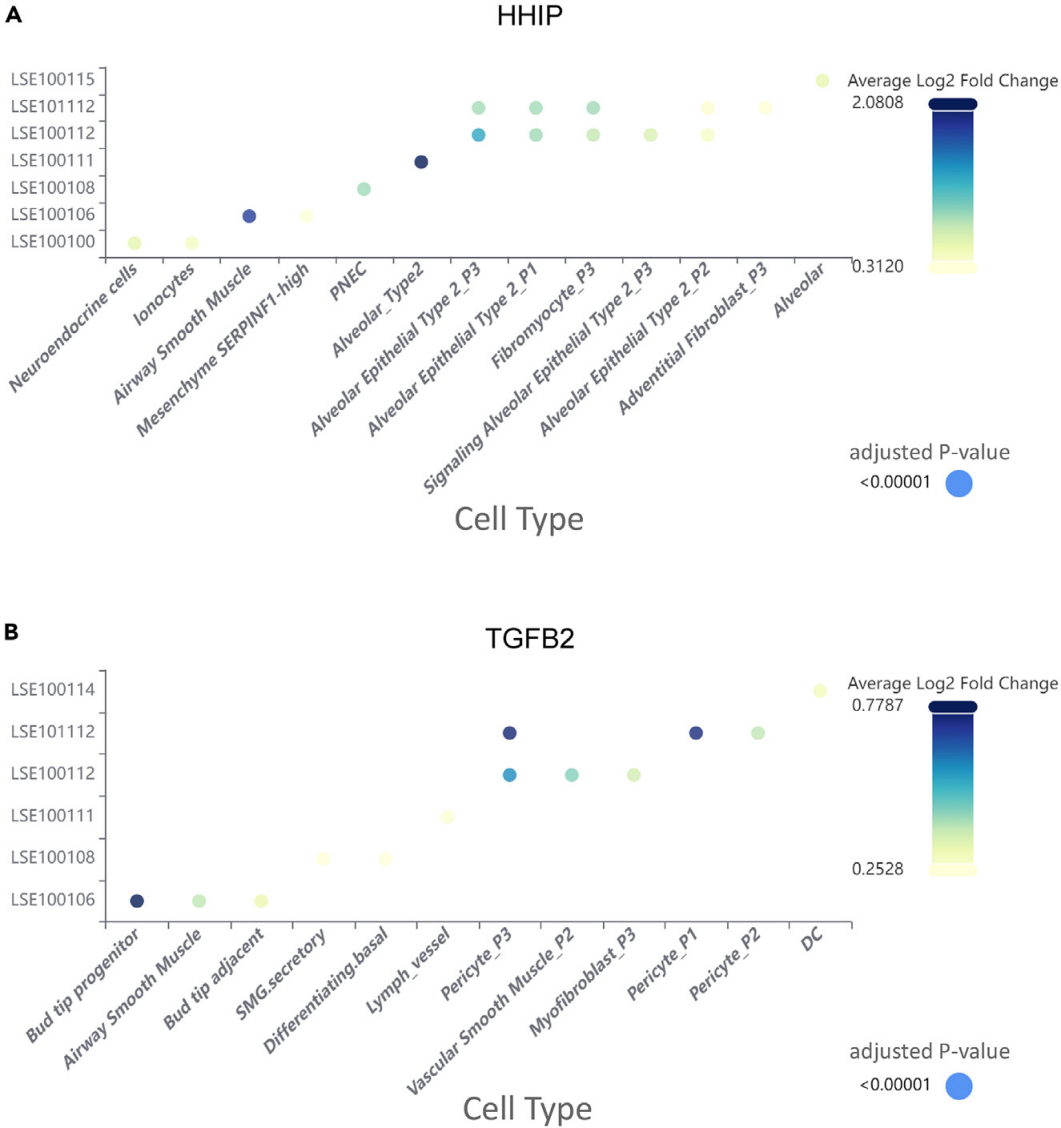
Differential expression of HHIP and TGFB2 across cell types and datasets Bubble plots delineating the differential expression of (A) HHIP and (B) TGFB2 genes across various cell types identified in multiple scRNA-seq datasets. Each data point specifies a cell type within a particular dataset, where the color intensity of the bubbles corresponds to the average log_2_ fold-change in gene expression, and the bubble size indicates the adjusted *p* value. Cell type markers were determined utilizing Wilcoxon rank-sum tests via the FindAllMarkers function in the Seurat package. Adjusted *p* value, based on Bonferroni correction using all genes in the dataset. Pulmonary neuroendocrine cell, PNEC; DC, dendritic cell.

**Figure 5:**
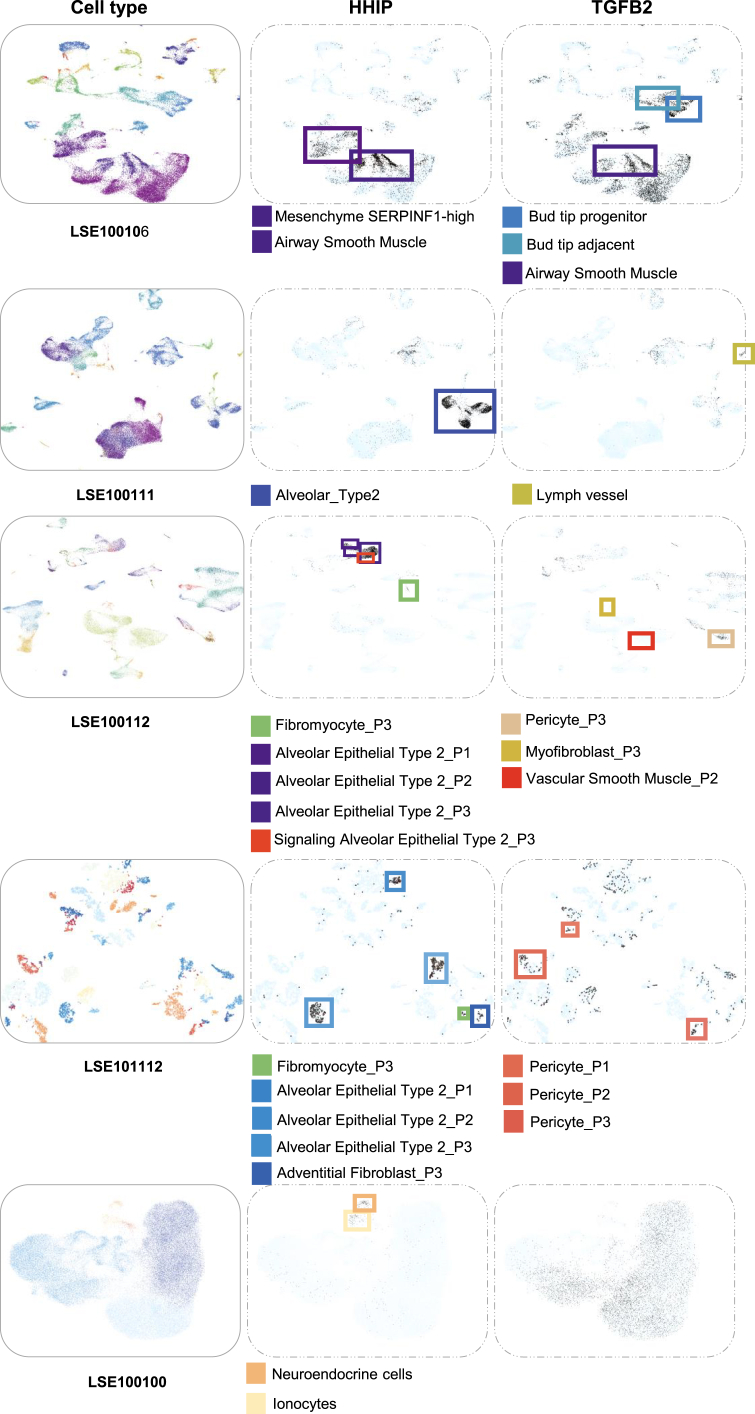
The UMAPs visualize cell types and their expressions of HHIP and TGFB2 in five datasets (dataset IDs: LSE100106, LSE100111, LSE100112, LSE101112, and LSE100100) The panels of 1^st^ column display cell types in UMAP plots with each cell type represented by a unique color. In the subsequent columns, the results of querying for HHIP (panels of 2^nd^ columns) and TGFB2 (panels of 3^rd^ columns) are illustrated with black plots marking the cells with high expressions of the query genes. The cell types that significantly express the query genes are enclosed by rectangle boxes in different colors. Pulmonary neuroendocrine cell, PNEC; DC, dendritic cell.

In pathology, COPD is characterized by airway remodeling,^31^ which refers to the thickening and deformation of airway wall due to cellular and structural changes. Previous research has linked the gene of Hedgehog-interacting protein (HHIP) through GWAS to the modulation of airway remodeling, revealing its role in curtailing the proliferation and metabolic reprogramming of airway smooth muscle cells.^32^ To validate this discovery and investigate the role of HHIP in diverse cellular contexts within the airway remodeling cascade, we firstly input the gene name of HHIP into the scMoresDB search box and conducted the search to yield a result table. Subsequently, accessing the bubble plots (Figure 4) through the provided link in the result table, we retrieved the cell types derived from multiple single-cell transcriptomics datasets. Employing conventional filtering criteria (log_2_ fold-change of average expression >0.25 and adjusted *p* value <1 × 10^−5^), we then retained the cell types with significantly high HHIP expression. Further exploration involved selecting one of the dataset IDs (e.g., LSE100106) in the result table, prompting scMoresDB to display the uniform manifold approximation and projections (UMAPs) in the Cell Browser (Figure 5). These UMAPs mark the cell types exhibiting significantly high HHIP expression.

The bubble plot (Figure 4A) indicates several key cell types with high HHIP expression, including adventitial fibroblasts, airway smooth muscle cells, alveolar epithelial cells, fibromyocytes, neuroendocrine cells, and ionocytes. High HHIP expression across multiple datasets (Figure 5: panels in 2^nd^ column) suggests potential implications of HHIP mutations on airway smooth muscle cells, possibly leading to smooth muscle hypertrophy.^32^ Additionally, these mutations might influence alveolar (epithelial) type II cells and fibromyocytes, ultimately resulting in destruction of alveolar wall and pulmonary fibrosis.^32^ The pathological changes of these cell types are associated with airway remodeling. Specifically, within the LSE100106 dataset, HHIP exhibits robust expression in airway smooth muscle cell (avg_log2_fc 1.82), aligning with prior studies.^32^ Concurrently, alveolar (epithelial) type II cells are notably prevalent in datasets LSE100111, LSE100112, and LSE101112, displaying relatively high HHIP expression compared to other cell types. This suggests that mutations in HHIP might significantly impact the function of alveolar (epithelial) type II cells, potentially contributing to surfactant insufficiency and alveolar instability in COPD.^33^ Moreover, fibromyocytes exhibiting heightened HHIP expression in datasets LSE100112 and LSE101112 may contribute to airway remodeling by differentiating into airway smooth muscle cells, leading to increased smooth muscle thickness.^34^ Regarding other cell types displaying high HHIP expression in datasets LSE101112 or LSE100100, neuroendocrine cell and ionocyte may contribute to mucous gland secretion,^35^^,^^36^ while adventitial fibroblast could be implicated in the fibrotic processes associated with COPD progression.

Emphysema, another pathological state of COPD that can arise from genetic perturbations, has been linked to TGFB2 gene using GWAS.^37^ Previous research has established a connection between TGFB2 and emphysema through its role in fibroblasts.^37^^,^^38^ In this study, we performed a similar search as described previously in scMoresDB to screen TGFB2 cell types in multiple scRNA-seq datasets using the same criteria (log_2_ fold-change of average expression >0.25, adjusted *p* value <1 × 10^−5^). The resulting bubble plot (Figure 4B) highlights key cell types with notable TGFB2 expression, including airway smooth muscle cells, bud tip adjacent cells, bud tip progenitor cells, dendritic cells, myofibroblasts, and pericytes. These cell types are predominantly mesenchymal components, potentially involved in processes, such as epithelial cell development, airway and vascular remodeling, excessive mucus secretion, and alveolar structural destruction.^31^ Specifically, high TGFB2 expression is observed in bud tip progenitor cells within dataset LSE100106 (Figure 5: top panel in 3^rd^ column). These lung progenitors significantly contribute to human lung development, repairing, and regeneration by differentiating into both alveolar and airway cells,^39^ suggesting a potential impact of TGFB2 mutations on airway remodeling. Concurrently, pericytes, prominently expressing TGFB2 in datasets LSE100112 and LSE101112 (Figure 5: 3^rd^ and 4^th^ panels in 3^rd^ column), are recognized for mediating endothelial proliferation and angiogenesis.^40^ Biopsies from COPD patients have revealed disrupted basement membranes, increased vascularization, and enhanced smooth muscle proliferation leading to thicker vascular walls.^41^ These findings implicate the contribution of TGFB2 in emphysema development through vascular remodeling.

**Figure 6:**
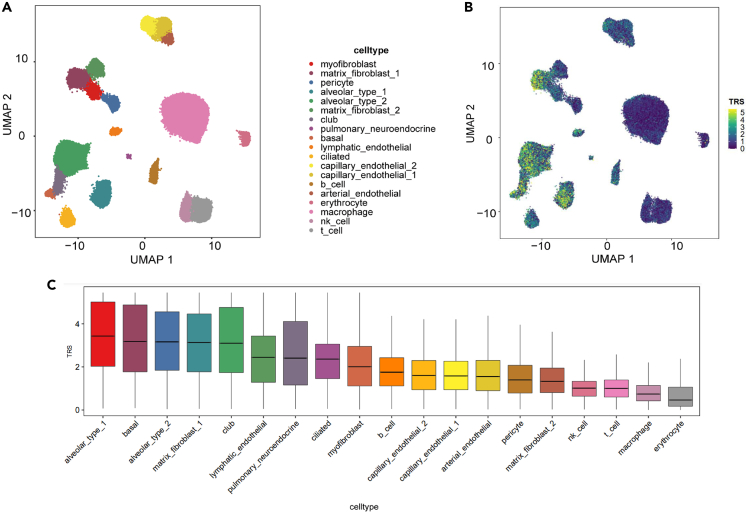
The analysis of disease-causal genetic variations of COPD at the single-cell resolution in scMoresDB (A) The UMAP representation of snATAC-seq of 90,980 cells from neonatal, pediatric, and adult samples with 19 cell types annotated. (B and C) TRS for the never-smoking COPD GWAS fine-mapping data displayed as (B) the UMAP plot and (C) the boxplot. Type I pneumocyte, alveolar_type_1; Type II pneumocyte, alveolar_type_2; Club cell, club; Ciliated cell, ciliated; Basal cell, basal; lymphatic endothelial cell, lymphatic_ endothelial; ciliated cell, ciliated; capillary endothelial cell, capillary_endothelial_2, capillary_endothelial_1; B cell, b_cell; arterial endothelial cell, arterial_endothelial; natural killer cell, nk_cell; T cell, t_cell.

To unravel how GWAS-identified risk loci drive disease progression, we incorporated the SCAVENGE tool into our scMoresDB. This specialized tool is designed to analyze single-nucleus ATAC-seq (snATAC-seq) data to assess enrichments of open chromatin regions associated with the risk loci. We proposed to combine the use of the snATAC-seq and GWAS data in scMoresDB to pinpoint cell types associated with COPD. Employing the SCAVENGE tool in scMoresDB, we correlated never-smoking COPD GWAS fine-mapping data with snATAC-seq data encompassing 90,980 profiles across 19 cell types (Figure 6A) from neonatal, pediatric, and adult samples. The output from SCAVENGE, termed the trait relevance score (TRS) for each single cell, reveals high TRS in certain cell types, such as basal cells, club cells, type I and type II pneumocytes, and matrix fibroblasts (Figures 6B and 6C), implying their associations with COPD. Notably, this result is highly consistent with the findings in Phuwanat Sakornsakolpat’s study,^42^ in which the prominent cell types (e.g., basal cell, club cell, fibroblasts, smooth muscle cell, and type II pneumocyte) are enriched in COPD heritability assessed through scRNA-seq.

## Discussion

scMoresDB gains significant advances in data coverage, database functionality, and research applications compared with other existing similar databases. In terms of data coverage, while databases like SC2diseases and Aging Atlas have been developed to accommodate large-scale single-cell sequencing data, they focus solely on single-cell transcriptomics data. On the other hand, the applications of scATAC-seq, scTCR-seq, CITE-seq, and spatial transcriptomic technologies have produced massive multimodal data, providing epigenetic, immunological, and spatial information to facilitate the inference of disease-associated expression regulations, cell types, and immune states. Existing databases like HTCA integrate diverse data types but are limited to healthy samples. Similarly, respiratory system-related databases, such as SCLC-CellMiner, LCMD, REALGAR, and ILDGDB, offer multi-omics and drug information but at bulk sequencing level, primarily focusing on a single disease of either lung cancer, asthma, or ILD. Notably, the LungMAP Consortium has established a series of network portals (e.g., LungMAP.net and LGEA) to provide single-cell, multi-omics, and imaging data. However, these data were mainly self-generated for consortium members. Furthermore, public datasets such as TCR-seq from immune receptor profiling offer potential usages in respiratory disease research. In comparison, our scMoresDB stands out by incorporating diverse data, spanning healthy and multi-disease samples, and integrating public single-cell datasets of 9 omics types. This broad scope and inclusivity make scMoresDB a comprehensive resource for respiratory disease investigation.

Regarding database functionality, scMoresDB undertakes dataset re-analysis through standard workflows and implements various tools for result visualization. The Cell Browser in scMoresDB allows user to easily visualize gene expression, chromatin accessibility, TCR clonotypes, and surface protein enrichment on a UMAP plot. Simultaneously, the Immune Abundance Browser provides detailed information on TRBV-TRBJ gene pairing and TCR sharing between cell types. Leveraging both browsers, we explored the immune cell status of COVID-19 patients in this study. Moreover, the Genome Browser enables comparative visualization between different datasets, such as chromatin accessibility and GWAS information, while the Spatial Browser permits examination of spatial gene expression distribution in tissue sections. For instance, we searched the BSG receptor of SARS-CoV-2 spike protein via the Spatial Browser and visualized its chromatin accessibility in promoter regions across different cell types as well as its spatial expression in lung tissue. The findings suggest the contribution of BSG in the process of SARS-CoV-2 infection. Additionally, we employed the volcano plot to visualize RNA-seq data for DEG analysis.

We conducted the analysis cases in COPD and COVID-19 to demonstrate the database application of scMoresDB for lung disease research. By integrating scRNA-seq, scATAC, and GWAS data in scMoresDB, we unveiled that the risk loci for COPD potentially affect the function of pulmonary epithelial cells and fibroblasts, heightening susceptibility to the disease. Notably, our analysis suggests that the risk loci in the HHIP gene identified by GWAS may be involved in the process of airway remodeling through their effects on type II alveolar cells. Additionally, the gene of TGFB2 appears to play a pivotal role in vascular remodeling. By combining scATAC, spatial transcriptomics, and bulk transcriptomics data, our study suggests that SARS-CoV-2 might invade alveolar epithelial cells through the BSG receptor, subsequently spreading to endothelial cells and immune cells. Moreover, by correlating scTCR-seq and scRNA-seq data, we found high clonal proportions in cytotoxic adaptive T cells within COVID-19 patients.

scMoresDB stands as a single-cell multi-omics database platform focusing on human respiratory system, boasting extensive omics types. It provides a user-friendly interface for seamless cross-omics data browsing and searching and integrates visualization and analysis tools for comprehensive data sharing and integrative analysis, thereby aiding researchers in better decoding respiratory single-cell omics data. Our example applications by the visualization tools hint at the potential significance of the BSG receptor in SARS-CoV-2 infection based on its high expression and potential interaction to alveolar epithelial cells. The case study in COPD suggests the involvement of HHIP and TGFB2 in the development and progression of COPD according to their expression changes at the single-cell resolution. Moreover, our findings propose that the risk loci for COPD could impact the function of pulmonary epithelial cells and fibroblasts, leading to more susceptibility to this disease. These discoveries offer valuable insights for the identification of research targets, indicating the value and importance of scMoresDB. Altogether, scMoresDB significantly increases the accessibility and utility of single-cell multi-omics data for human respiratory system and associated diseases, accelerating data analysis and applications in both biological and clinical research.

### Limitations of the study

This study has certain limitations. scMoresDB offers a singular online tool for the integrative analysis of GWAS and scATAC-seq. While our intent is to include more tools for integrative multi-omics data analysis, the current scarcity of such tools within the research community impedes this endeavor. In addition, we analyzed each dataset independently and extracted information by matching the same keywords. To some extent, the effectiveness of this approach relies on the prior knowledge of the user. In the future, knowledge bases can be built to strengthen the connection between information of different datasets and thus to discover new biological insights. Furthermore, the application instances highlighted in this study primarily serve to illustrate the visualization techniques and the utility of the online analysis tool. It is important to acknowledge that the findings derived from the case studies lack comprehensive experimental verification.

## STAR★Methods

### Key resources table

**Table:** 

REAGENT or RESOURCE	SOURCE	IDENTIFIER
**Software and algorithms**		

R 4.0.3	R Core Team	https://www.r-project.org
Seurat 4.1.0	Hao et al., 2021^43^	https://satijalab.org/seurat/
Signac	Stuart et al., 2021^44^	https://stuartlab.org/signac/
UCSC Cell Browser	Speir et al., 2021^45^	https://github.com/maximilianh/cellBrowser
Sinto	https://github.com/timoast/sinto	https://github.com/timoast/sinto
STUtility	Bergenstråhle et al., 2020^46^	https://github.com/jbergenstrahle/STUtility
AUCell	Aibar et al., 2017^47^	https://github.com/aertslab/AUCell
ChIPseeker	Wang et al., 2022^48^	https://github.com/YuLab-SMU/ChIPseeker
Limma	Ritchie et al., 2015^49^	https://bioinf.wehi.edu.au/limma/
scRepertoire	Borcherding et al., 2020^50^	https://github.com/ncborcherding/scRepertoire
SpringBoot 2.5.6	https://spring.io/projects/spring-boot	https://spring.io/projects/spring-boot
Vue.js 2.0	https://vuejs.org/	https://vuejs.org/
MySQL 5.7	https://www.mysql.com	https://www.mysql.com
igv.js 2.13.3	Robinson et al., 2023^26^	https://github.com/igvteam/igv.js/
HTTPD 2.4.6	https://httpd.apache.org/	https://httpd.apache.org/
Tomcat 9.0.54	https://tomcat.apache.org/	https://tomcat.apache.org/
HighCharts 10.3.2	https://www.hcharts.cn	https://www.hcharts.cn
Shiny 1.5.18	https://shiny.posit.co/	https://shiny.posit.co/
VolcanNoseR 1.0.3	Goedhart and Luijsterburg, 2020^25^	https://github.com/JoachimGoedhart/VolcaNoseR

**Other**		

Resource website for the publication	This paper	http://www.liwzlab.cn/scmoresdb

### Resource availability

#### Lead contact

Further information and requests for resources should be directed to and will be fulfilled by the Lead Contact, Weizhong Li (liweizhong@mail.sysu.edu.cn).

#### Materials availability

This study did not generate new unique reagents.

#### Data and code availability

• All public source data used in this study were listed in the Table S1. All analysis results are accessible through the search pages of scMoresDB (http://www.liwzlab.cn/scmoresdb/#/Browse).
• The scripts used in data processing can be got through reasonable requests.
• Any additional information required to reanalyse the data reported in this paper is available from the lead contact (Weizhong Li, liweizhong@mail.sysu.edu.cn) upon request.

### Method details

#### Source data integration

The source datasets were collected from GEO,^51^ ArrayExpress,^52^ UCSC Cell Browser,^45^ immuneACCESS (https://clients.adaptivebiotech.com), Single Cell Portal (https://singlecell.broadinstitute.org/single_cell), Mendeley Data (https://data.mendeley.com), TCRdb,^53^ CAUSALdb,^54^ GWAS Catalog,^55^ and several publication-related web repositories^56^^,^^57^ (Figure 2A) (Tables 1 and S1), followed by data filtering and normalization under standard quality control protocols, as well as data integration, clustering, and annotation by prevalent tools (Figure 2B). Details about the data pre-processing and parameter settings for each dataset can be found on the summary tab of the dataset visualization page, for instance http://www.liwzlab.cn/scmoresdb/#/Visualization/LSE100100.

To ensure uniform search and visualization through the web application, we employed specific data processing pipelines for different types of omics data. In details, the scRNA-seq datasets were initially pre-processed by Seurat (v4.1.0)^43^ for quality control, normalization, dimension reduction, clustering, and cell-type annotation according to relevant publications and metadata (Figure 2B). Subsequently, the expressions of differentially expressed genes (DEGs) were computed across various cell types using Wilcoxon test, as implemented in Seurat's FindAllMarkers function. The DEG criteria included a log-scale fold difference > 0.25, a min.pct threshold of 0.1, and a min.diff.pct > 0.05. Seurat objects were then saved and transferred to the CellBrowser web application using the UCSC Cell Browser functions ‘cbSeuratImport’ and ‘cbBuild’.

Similarly, the datasets of scATAC-seq, CITE-seq, and spatial transcriptomics were processed by Seurat and Signac^44^ for data pre-processing, cell-type annotation, and DEGs identification (Figure 2B). Particularly, we created a gene activity matrix for scATAC-seq before cell-type annotation and adopted Sinto (https://github.com/timoast/sinto) to split the tracks of different cell types for visualization in the Genome Browser. Meanwhile, we retrieved and processed the information of RNAs, hashtag oligos (HTOs), and antibody-derived tags (ADTs) separately from CITE-seq data, and integrated multimodal information before cell-type annotation. For spatial transcriptomic data, we further annotated genes that are highly varied in space by STUtility^46^ and AUCell^47^ (Figure 2B). The same parameter setting for scRNA-seq data pre-processing was employed to compute expressions of DEGs and ADT profiles for CITE-seq. For scATAC-seq data, differentially accessible chromatin regions across various cell types were annotated using Chipseeker.^48^ The Seurat function ‘FindSpatiallyVariableFeatures’ was applied to find spatial DEGs across cell clusters generated by Seurat, and the resulting Seurat objects were saved for further spatial analysis and visualization. For CITE-seq and scATAC-seq data, we used the applications ‘cbSeuratImport’ and ‘cbBuild’ in CellBrowser to export gene expressions and ADT matrices as well as accessible chromatin regions matrix, respectively. In the case of spatial transcriptomics, Seurat objects were utilized by the application Shiny for visualization.

The bulk gene expression datasets containing RNA-seq and microarray data were normalized. RNA-seq data was uniformly converted to transcripts in millions (TPM), and Wilcoxon test was performed to find DEGs based on the experimental design of control and treatment groups. Meanwhile, batch effects were removed by limma^49^ for microarray datasets, followed by a differential expression analysis using a linear model (Figure 2B).

We annotated the datasets of bulk ATAC-seq and genome-wide association studies (GWAS) using ChIPseeker (Figure 2B). ATAC-seq datasets have been processed from MACS2^58^ peak calling before downloading. Meanwhile, the downloaded GWAS datasets have been undergone for fine mapping processes. Hence, we were able to directly annotate the mapped genes of chromosome positions, along with their upstream and downstream information. Promoters were defined as regions within 2000 base pairs upstream and downstream of the transcription start site (Figure 2B).

For scTCR-seq data, we used scRepertoire^50^ to merge contigs and integrate them with the processed scRNA-seq data from the same single cells (Figure 2B). Then the common clonotypes among different groups and clusters were generated for visualisation in the Immune Abundance Browser. Complete TCR sequences were preserved from bulk TCR-seq datasets, retaining key information of V gene, J gene, and in-frame complementarity determining region 3 (CDR3) (Figure 2B).

#### Web implementation

The scMoresDB website, implemented in Java language and the SpringBoot (v2.5.6) framework, was deployed by Apache web server. The frontend interface was visualized by using the Vue.js framework (v2.0). All data were stored in a MySQL database (v5.7). The analysis tools were implemented using in-house R scripts. The Cell Browser and the Genome Browser, integrated based on UCSC Cell Browser and igv.js, were deployed by HTTPD (v2.4.6) and Tomcat (v9.0.54) servers, respectively. The Immune Abundance Browser was developed using HighCharts (v10.3.2). Shiny (v1.5.18) and VolcanNoseR^25^ were exploited for the Spatial Browser and volcano plotting (Figure 2C).

### Quantification and statistical analysis

For the analyses of scRNA-seq, scATAC-seq, CITE-seq, and spatial transcriptome, we conducted the Wilcoxon rank-sum test using the FindAllMarkers function in the Seurat software package (version 4.1.0). For our case study in COPD, we focused on data with a log2 fold-change in average expression over 0.25 and an adjusted P-value below 1×10^-5^. The number of cells in each dataset, which represents the sample size, can be found in the dataset description on the database website. We standardized the RNA sequencing data to TPM and then used the Wilcoxon rank-sum test again to identify DEGs under different conditions. For the microarray datasets, we corrected batch effects using the limma tool and then performed differential expression analysis using a linear model. We applied the Bonferroni correction method to minimize false positives to increase the statistical accuracy for the above results.

## Supplemental information

Table S1. The data source of scMoresDB, related to Figure 2
